# Supplementary material for: Coarse-Grained Molecular Dynamics Simulations of Lipid Nanodroplets and Endosomal Membranes: Focusing on the Fusion Mechanisms
Source: Int J Mol Sci. 2025 Dec 11;26(24):11960. doi: 10.3390/ijms262411960 (PMC12732718; doi:10.3390/ijms262411960)
Supplement: Supplementary file 1 [file ijms-26-11960-s001.zip › ijms-3960198-supplementary.pdf]

Supporting Information for

Coarse-grained molecular dynamics simulations of lipid nano droplets and endosomal membranes: Focusing on the fusion mechanism

Yeon Ju Go, Erkhembayar Jadamba,* and Hyunjin Shin*

MOGAM Institute for Biomedical Research (MIBR),

Seoul 06730, Republic of Korea

Table S1. Rates of protonated (P) and deprotonated (D) ALC-0315 migrating to the outer leaflet and membrane midplane during the fusion of LNDs with EEM or LEM, based on three independent replicas.

Protonated case (%)	Outer leaflet ratio (P)	Outer leaflet ratio (D)	Midplane ratio (P)	Midplane ratio (D)
0	–	0.06 ± 0.03	–	0.88 ± 0.04
0	–	0.09 ± 0.03	–	0.87 ± 0.04
0	–	0.08 ± 0.03	–	0.87 ± 0.04
10	0.39 ± 0.14	0.06 ± 0.03	0.45 ± 0.18	0.89 ± 0.04
10	0.23 ± 0.16	0.06 ± 0.03	0.48 ± 0.16	0.89 ± 0.04
10	0.49 ± 0.13	0.06 ± 0.03	0.30 ± 0.21	0.89 ± 0.05
20	0.21 ± 0.10	0.05 ± 0.03	0.40 ± 0.13	0.90 ± 0.04
20	0.29 ± 0.11	0.05 ± 0.03	0.38 ± 0.11	0.91 ± 0.05
20	0.47 ± 0.14	0.07 ± 0.04	0.39 ± 0.13	0.89 ± 0.04
30	0.29 ± 0.09	0.05 ± 0.04	0.43 ± 0.10	0.92 ± 0.04
30	0.35 ± 0.07	0.05 ± 0.03	0.47 ± 0.10	0.92 ± 0.04
30	0.32 ± 0.07	0.04 ± 0.03	0.42 ± 0.10	0.93 ± 0.04
40	0.36 ± 0.07	0.03 ± 0.03	0.37 ± 0.08	0.94 ± 0.04
40	0.25 ± 0.06	0.04 ± 0.04	0.46 ± 0.08	0.91 ± 0.05
40	0.34 ± 0.10	0.04 ± 0.03	0.40 ± 0.09	0.94 ± 0.04
50	0.27 ± 0.07	0.03 ± 0.03	0.51 ± 0.08	0.95 ± 0.04
50	0.32 ± 0.05	0.03 ± 0.03	0.44 ± 0.07	0.95 ± 0.04
50	0.27 ± 0.05	0.05 ± 0.04	0.43 ± 0.06	0.92 ± 0.05
60	0.27 ± 0.05	0.04 ± 0.04	0.46 ± 0.07	0.95 ± 0.04
60	0.30 ± 0.06	0.03 ± 0.03	0.47 ± 0.06	0.95 ± 0.05
60	0.23 ± 0.05	0.02 ± 0.03	0.48 ± 0.06	0.95 ± 0.04
70	0.35 ± 0.05/<br>0.29 ± 0.05	0.01 ± 0.03/<br>0.03 ± 0.04	0.49 ± 0.06/<br>0.30 ± 0.06	0.98 ± 0.04/<br>0.93 ± 0.06
70	0.29 ± 0.05/<br>0.37 ± 0.05	0.04 ± 0.04/<br>0.05 ± 0.06	0.48 ± 0.06/<br>0.33 ± 0.05	0.95 ± 0.05/<br>0.92 ± 0.07
70	0.30 ± 0.04/<br>0.23 ± 0.05	0.01 ± 0.03/<br>0.03 ± 0.04	0.48 ± 0.06/<br>0.36 ± 0.08	0.98 ± 0.04/<br>0.92 ± 0.08
80	0.46 ± 0.05	0.04 ± 0.05	0.32 ± 0.05	0.94 ± 0.06
80	0.39 ± 0.05	0.03 ± 0.06	0.33 ± 0.06	0.93 ± 0.08
80	0.30 ± 0.06	0.04 ± 0.06	0.33 ± 0.07	0.94 ± 0.07
90	0.48 ± 0.05	0.15 ± 0.16	0.14 ± 0.06	0.71 ± 0.21
90	0.44 ± 0.05	0.14 ± 0.16	0.15 ± 0.07	0.70 ± 0.23
90	0.30 ± 0.05	0.03 ± 0.07	0.18 ± 0.05	0.54 ± 0.21

100	0.51 ± 0.04	–	0.09 ± 0.04	–
100	0.41 ± 0.04	–	0.10 ± 0.04	–
100	0.40 ± 0.05	–	0.10 ± 0.04	–

Table S2. Migration rates of ALC-0315 to the outer leaflet and membrane midplane during the fusion of LNDs with SLB, obtained from three independent replicas.

Protonated case (%)	Outer leaflet ratio (P)	Outer leaflet ratio (D)	Midplane ratio (P)	Midplane ratio (D)
0	–	0.17 ± 0.04	–	0.71 ± 0.06
0	–	0.09 ± 0.03	–	0.71 ± 0.05
0	–	0.15 ± 0.04	–	0.76 ± 0.05
10	0.62 ± 0.17	0.14 ± 0.04	0.08 ± 0.12	0.72 ± 0.06
10	0.48 ± 0.14	0.16 ± 0.04	0.16 ± 0.13	0.73 ± 0.06
10	0.27 ± 0.17	0.07 ± 0.03	0.17 ± 0.18	0.71 ± 0.06
20	0.61 ± 0.11	0.10 ± 0.04	0.19 ± 0.09	0.74 ± 0.06
20	0.42 ± 0.21	0.22 ± 0.06	0.15 ± 0.11	0.70 ± 0.06
20	0.43 ± 0.09	0.13 ± 0.05	0.18 ± 0.09	0.76 ± 0.06
30	0.61 ± 0.07	0.13 ± 0.05	0.08 ± 0.07	0.76 ± 0.07
30	0.65 ± 0.09	0.17 ± 0.05	0.13 ± 0.08	0.75 ± 0.06
30	0.52 ± 0.07	0.13 ± 0.04	0.17 ± 0.09	0.77 ± 0.05
40	0.32 ± 0.08	0.10 ± 0.06	0.09 ± 0.07	0.75 ± 0.08
40	0.54 ± 0.07	0.12 ± 0.06	0.13 ± 0.06	0.77 ± 0.09
40	0.55 ± 0.07	0.21 ± 0.07	0.09 ± 0.06	0.69 ± 0.08
50	0.42 ± 0.07	0.12 ± 0.07	0.07 ± 0.05	0.65 ± 0.13
50	0.50 ± 0.05	0.11 ± 0.06	0.11 ± 0.06	0.81 ± 0.08
50	0.40 ± 0.06	0.10 ± 0.05	0.15 ± 0.07	0.79 ± 0.07
60	0.38 ± 0.06	0.15 ± 0.08	0.11 ± 0.07	0.65 ± 0.14
60	0.50 ± 0.06	0.27 ± 0.11	0.06 ± 0.04	0.56 ± 0.14
60	0.54 ± 0.04	0.23 ± 0.09	0.06 ± 0.04	0.57 ± 0.13
70	0.61 ± 0.05	0.27 ± 0.11	0.06 ± 0.04	0.55 ± 0.15
70	0.42 ± 0.05	0.25 ± 0.10	0.06 ± 0.04	0.48 ± 0.12
70	0.68 ± 0.05	0.42 ± 0.11	0.06 ± 0.04	0.46 ± 0.13
80	0.56 ± 0.04	0.33 ± 0.14	0.06 ± 0.04	0.48 ± 0.16
80	0.51 ± 0.04	0.22 ± 0.12	0.06 ± 0.04	0.45 ± 0.17
80	0.47 ± 0.06	0.26 ± 0.14	0.05 ± 0.04	0.47 ± 0.16
90	0.52 ± 0.04	0.33 ± 0.20	0.05 ± 0.03	0.49 ± 0.23
90	0.52 ± 0.03	0.32 ± 0.20	0.06 ± 0.03	0.47 ± 0.23
90	0.44 ± 0.03	0.38 ± 0.21	0.06 ± 0.04	0.44 ± 0.23
100	0.47 ± 0.03	–	0.06 ± 0.03	–
100	0.55 ± 0.04	–	0.06 ± 0.03	–
100	0.55 ± 0.03	–	0.06 ± 0.03	–

Table S3. Rates of MC3 migrating to the outer leaflet and membrane midplane during LND fusion with EEM or LEM, based on three independent replicas.

Protonated case (%)	Outer leaflet ratio (P)	Outer leaflet ratio (D)	Midplane ratio (P)	Midplane ratio (D)
0	–	0.06 ± 0.03	–	0.90 ± 0.04
0	–	0.06 ± 0.03	–	0.91 ± 0.04
0	–	0.04 ± 0.02	–	0.90 ± 0.04
10	0.48 ± 0.16	0.05 ± 0.03	0.24 ± 0.18	0.92 ± 0.04
10	0.55 ± 0.21	0.05 ± 0.03	0.13 ± 0.15	0.91 ± 0.04
10	0.25 ± 0.13	0.04 ± 0.03	0.22 ± 0.17	0.92 ± 0.04
20	0.34 ± 0.11	0.04 ± 0.03	0.20 ± 0.12	0.93 ± 0.04
20	0.45 ± 0.10	0.04 ± 0.03	0.21 ± 0.12	0.93 ± 0.04
20	0.35 ± 0.12	0.05 ± 0.03	0.25 ± 0.12	0.93 ± 0.04
30	0.44 ± 0.08	0.04 ± 0.03	0.23 ± 0.10	0.92 ± 0.04
30	0.46 ± 0.09	0.04 ± 0.03	0.26 ± 0.11	0.92 ± 0.04
30	0.37 ± 0.09	0.03 ± 0.03	0.21 ± 0.09	0.94 ± 0.04
40	0.45 ± 0.08	0.04 ± 0.04	0.25 ± 0.09	0.94 ± 0.05
40	0.33 ± 0.07	0.04 ± 0.04	0.23 ± 0.09	0.94 ± 0.05
40	0.36 ± 0.07	0.04 ± 0.03	0.23 ± 0.08	0.94 ± 0.04
50	0.34 ± 0.07	0.04 ± 0.04	0.29 ± 0.08	0.93 ± 0.05
50	0.44 ± 0.07	0.03 ± 0.03	0.28 ± 0.09	0.95 ± 0.04
50	0.46 ± 0.06	0.03 ± 0.03	0.29 ± 0.07	0.95 ± 0.04
60	0.48 ± 0.06	0.02 ± 0.03	0.28 ± 0.07	0.96 ± 0.04
60	0.42 ± 0.05	0.02 ± 0.03	0.28 ± 0.07	0.95 ± 0.05
60	0.34 ± 0.07	0.03 ± 0.04	0.23 ± 0.07	0.95 ± 0.05
70	0.41 ± 0.06	0.02 ± 0.04	0.28 ± 0.07	0.96 ± 0.05
70	0.40 ± 0.06	0.03 ± 0.04	0.26 ± 0.06	0.96 ± 0.05
70	0.32 ± 0.06	0.02 ± 0.03	0.31 ± 0.07	0.96 ± 0.05
80	0.38 ± 0.05	0.01 ± 0.04	0.29 ± 0.06	0.98 ± 0.05
80	0.30 ± 0.04	0.01 ± 0.03	0.29 ± 0.06	0.98 ± 0.04
80	0.38 ± 0.05	0.01 ± 0.03	0.29 ± 0.07	0.97 ± 0.05
90	0.56 ± 0.04	0.11 ± 0.14	0.06 ± 0.04	0.82 ± 0.17
90	0.54 ± 0.03	0.12 ± 0.14	0.07 ± 0.04	0.78 ± 0.19
90	0.54 ± 0.04	0.12 ± 0.13	0.07 ± 0.04	0.76 ± 0.20
100	0.39 ± 0.04	–	0.06 ± 0.04	–
100	0.40 ± 0.04	–	0.07 ± 0.03	–
100	0.34 ± 0.04	–	0.07 ± 0.03	–

Table S4. Rates of MC3 migrating to the outer leaflet and membrane midplane during fusion of LNDs with SLB, based on three independent replicas.

Protonated case (%)	Outer leaflet ratio (P)	Outer leaflet ratio (D)	Midplane ratio (P)	Midplane ratio (D)
0	–	0.06 ± 0.03	–	0.73 ± 0.06
0	–	0.16 ± 0.05	–	0.73 ± 0.06
0	–	0.16 ± 0.04	–	0.72 ± 0.06
10	0.17 ± 0.07	0.13 ± 0.04	0.05 ± 0.10	0.73 ± 0.06
10	0.33 ± 0.11	0.17 ± 0.05	0.08 ± 0.12	0.75 ± 0.07
10	0.43 ± 0.13	0.12 ± 0.05	0.04 ± 0.09	0.75 ± 0.06
20	0.40 ± 0.10	0.16 ± 0.06	0.06 ± 0.07	0.74 ± 0.07
20	0.55 ± 0.08	0.15 ± 0.05	0.04 ± 0.06	0.74 ± 0.07
20	0.47 ± 0.07	0.10 ± 0.04	0.06 ± 0.07	0.76 ± 0.07
30	0.64 ± 0.07	0.19 ± 0.06	0.06 ± 0.06	0.73 ± 0.07
30	0.35 ± 0.06	0.14 ± 0.06	0.05 ± 0.05	0.74 ± 0.07
30	0.75 ± 0.06	0.13 ± 0.05	0.04 ± 0.05	0.74 ± 0.08
40	0.41 ± 0.04	0.21 ± 0.07	0.05 ± 0.05	0.71 ± 0.08
40	0.45 ± 0.07	0.12 ± 0.06	0.05 ± 0.05	0.75 ± 0.08
40	0.46 ± 0.11	0.17 ± 0.06	0.05 ± 0.05	0.74 ± 0.08
50	0.51 ± 0.05	0.16 ± 0.07	0.05 ± 0.04	0.73 ± 0.09
50	0.49 ± 0.05	0.16 ± 0.07	0.05 ± 0.04	0.73 ± 0.09
50	0.52 ± 0.04	0.18 ± 0.07	0.05 ± 0.04	0.72 ± 0.09
60	0.39 ± 0.08	0.22 ± 0.09	0.05 ± 0.04	0.71 ± 0.10
60	0.44 ± 0.06	0.11 ± 0.07	0.05 ± 0.04	0.74 ± 0.11
60	0.58 ± 0.04	0.15 ± 0.07	0.05 ± 0.04	0.73 ± 0.10
70	0.58 ± 0.05	0.18 ± 0.10	0.05 ± 0.04	0.73 ± 0.12
70	0.59 ± 0.04	0.18 ± 0.10	0.05 ± 0.04	0.71 ± 0.12
70	0.52 ± 0.05	0.19 ± 0.10	0.05 ± 0.04	0.72 ± 0.11
80	0.59 ± 0.04	0.17 ± 0.12	0.05 ± 0.03	0.72 ± 0.14
80	0.59 ± 0.04	0.19 ± 0.12	0.05 ± 0.04	0.71 ± 0.14
80	0.49 ± 0.06	0.15 ± 0.11	0.04 ± 0.03	0.72 ± 0.14
90	0.54 ± 0.03	0.12 ± 0.14	0.04 ± 0.03	0.71 ± 0.21
90	0.60 ± 0.06	0.22 ± 0.18	0.05 ± 0.03	0.69 ± 0.21
90	0.57 ± 0.03	0.14 ± 0.15	0.05 ± 0.03	0.71 ± 0.21
100	0.56 ± 0.03	–	0.05 ± 0.03	–
100	0.55 ± 0.04	–	0.05 ± 0.03	–
100	0.45 ± 0.04	–	0.05 ± 0.03	–

Table S5. Percentages of protonated and deprotonated MC3 in the outer leaflet and midplane during the fusion of MC3–containing LNDs with EEM or LEM, detailed by protonated case.

Protonated case (%)	Outer leaflet ratio (P)	Midplane ratio (P)	Outer leaflet ratio (D)	Midplane ratio (D)
0	–	–	0.05 ± 0.02	0.90 ± 0.02
10	0.43 ± 0.10	0.20 ± 0.10	0.05 ± 0.02	0.91 ± 0.02
20	0.38 ± 0.06	0.22 ± 0.07	0.04 ± 0.02	0.93 ± 0.02
30	0.42 ± 0.05	0.23 ± 0.06	0.04 ± 0.02	0.92 ± 0.02
40	0.38 ± 0.04	0.24 ± 0.05	0.04 ± 0.02	0.94 ± 0.03
50	0.42 ± 0.04	0.29 ± 0.05	0.03 ± 0.02	0.95 ± 0.03
60	0.41 ± 0.03	0.26 ± 0.04	0.03 ± 0.02	0.96 ± 0.03
70	0.38 ± 0.03	0.28 ± 0.04	0.02 ± 0.02	0.96 ± 0.03
80	0.35 ± 0.03	0.29 ± 0.03	0.01 ± 0.02	0.97 ± 0.03
90	0.55 ± 0.02	0.07 ± 0.02	0.12 ± 0.08	0.79 ± 0.11
100	0.38 ± 0.02	0.07 ± 0.02	–	–

Table S6. Protonated and deprotonated MC3 ratios in the outer leaflet and midplane during the fusion of MC3–containing LNDs with SLB, detailed by protonated case.

Protonated case (%)	Outer leaflet ratio (P)	Midplane ratio (P)	Outer leaflet ratio (D)	Midplane ratio (D)
0	–	–	0.13 ± 0.02	0.73 ± 0.04
10	0.31 ± 0.06	0.06 ± 0.06	0.14 ± 0.03	0.75 ± 0.04
20	0.47 ± 0.05	0.05 ± 0.04	0.14 ± 0.03	0.74 ± 0.04
30	0.58 ± 0.04	0.05 ± 0.03	0.15 ± 0.03	0.74 ± 0.04
40	0.44 ± 0.04	0.05 ± 0.03	0.17 ± 0.04	0.73 ± 0.05
50	0.51 ± 0.03	0.05 ± 0.02	0.17 ± 0.04	0.73 ± 0.05
60	0.47 ± 0.04	0.05 ± 0.02	0.16 ± 0.04	0.72 ± 0.06
70	0.56 ± 0.03	0.05 ± 0.02	0.19 ± 0.06	0.72 ± 0.07
80	0.56 ± 0.03	0.05 ± 0.02	0.17 ± 0.07	0.72 ± 0.08
90	0.57 ± 0.02	0.05 ± 0.02	0.16 ± 0.09	0.71 ± 0.12
100	0.52 ± 0.02	0.05 ± 0.02	–	–

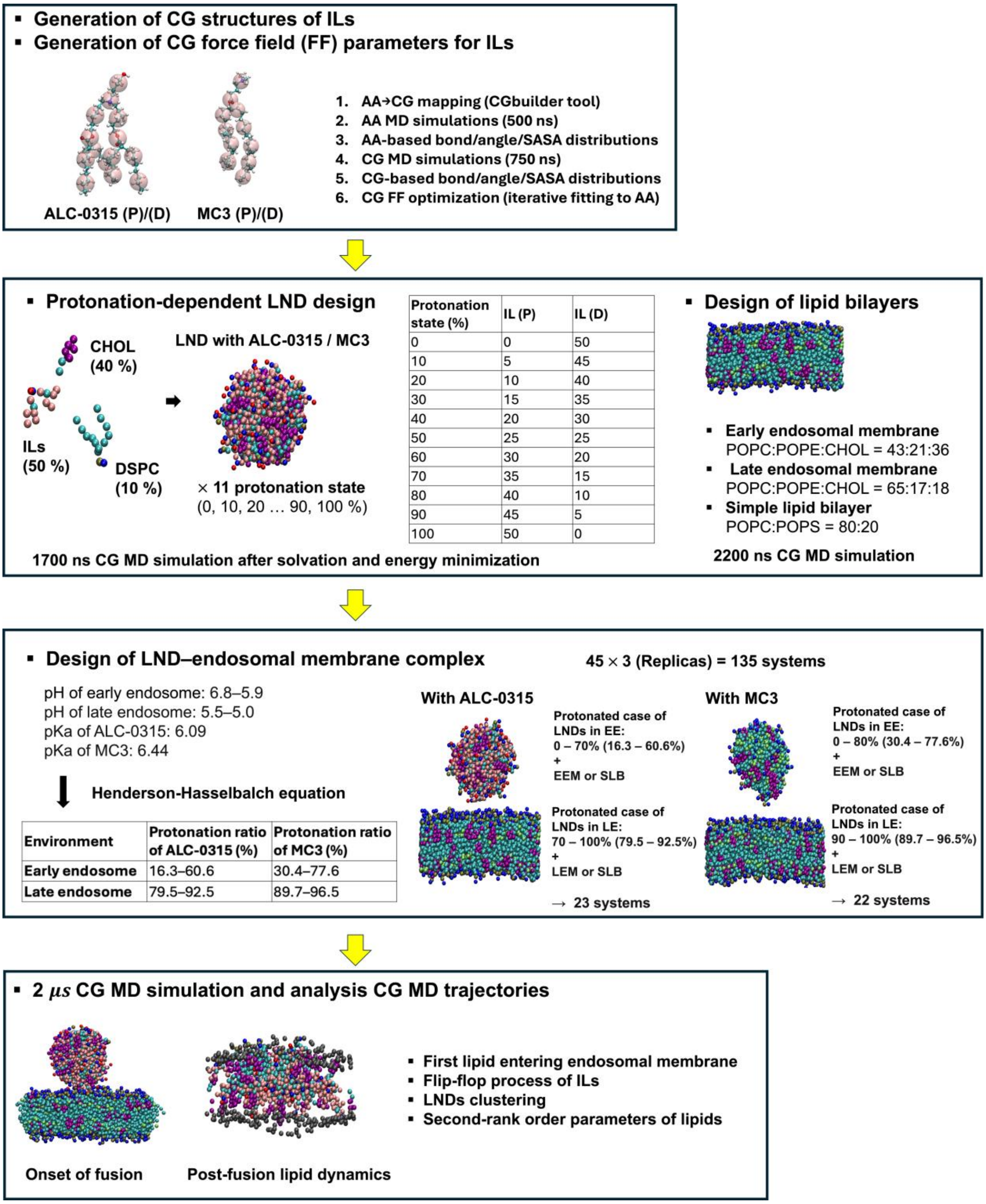

Figure S1. A detailed illustration of the computational steps, including the generation of CG structures and force field parameters for ILs, protonation-dependent LND design, construction of lipid bilayers and LND–endosomal membrane complexes, and CG MD simulations followed by trajectory analysis.

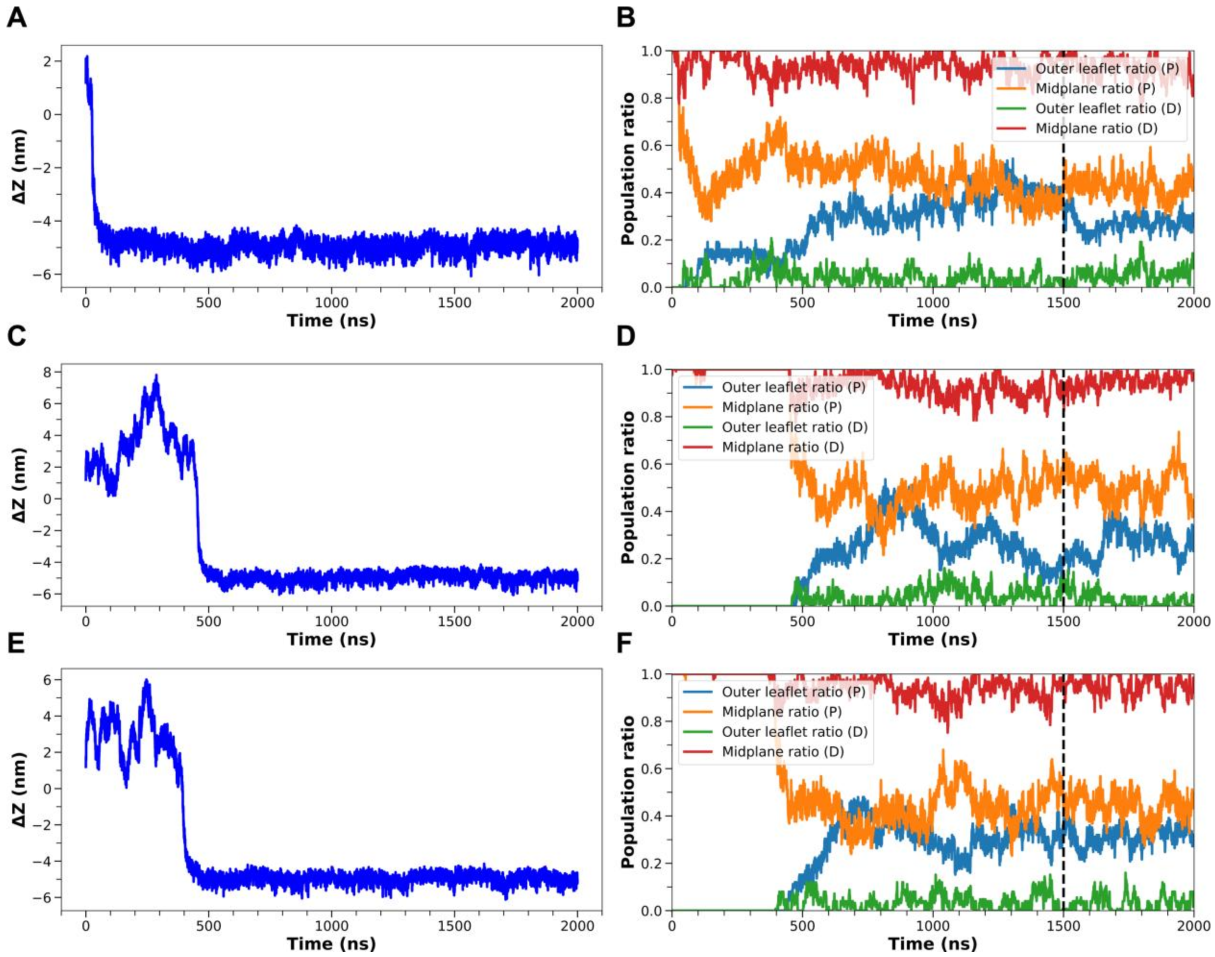

Figure S2. Time-dependent behavior of fusion onset (ΔZ) and ALC-0315 migration fractions for an LND containing 50% protonated ALC-0315 fused with EEM. (A) Fusion onset (ΔZ) for replica 1. (B) Migration fractions of ALC-0315 (P) and ALC-0315 (D) for replica 1: protonated ILs in the outer leaflet (blue) and midplane (orange), and deprotonated ILs in the outer leaflet (green) and midplane (red). (C) Fusion onset (ΔZ) for replica 2. (D) Migration fractions of ALC-0315 (P) and ALC-0315 (D) for replica 2. (E) Fusion onset (ΔZ) for replica 3. (F) Migration fractions of ALC-0315 (P) and ALC-0315 (D) for replica 3.

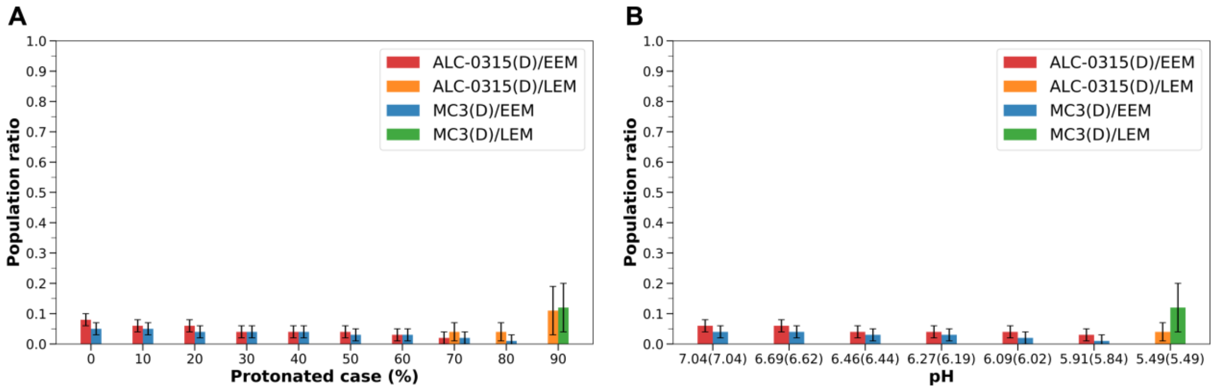

Figure S3. (A) Population ratios of ALC-0315(D) and MC3(D) in the outer leaflet during the fusion of ALC-0315-containing and MC3-containing LNDs with EEM or LEM, according to the LNDs' protonated case. Red and orange bars indicate the ratio for ALC-0315(D) when fusing with EEM and LEM, respectively. Blue and green bars show the ratio for MC3(D) when fusing with EEM and LEM, respectively. (B) Population ratios of ALC-0315(D) and MC3(D) in the outer leaflet, shown according to the pH of the LNDs.

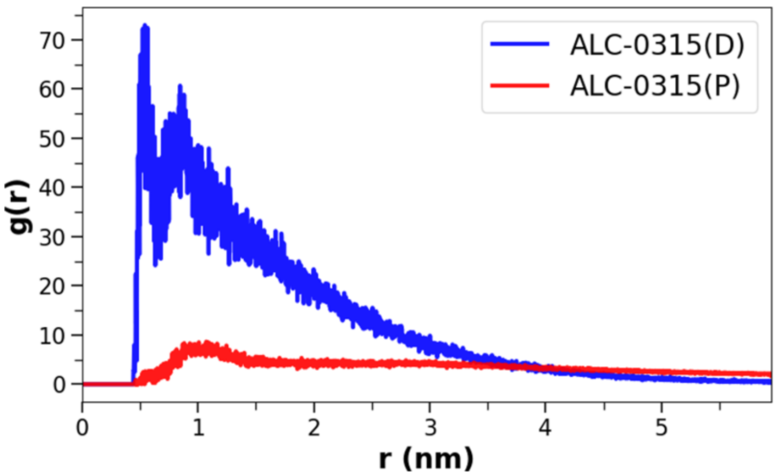

Figure S4. RDFs, g(r) of NC3 CG beads for ALC-0315(P) and ALC-0315(D) after the fusion of LNDs with 70% protonated ALC-0315 and LEM. The variable r indicates the distance between two beads. ALC-0315(P) is illustrated in red, and ALC-0315(D) in blue.

CG force field parameterization for ALC-0315 and MC3

All atomistic molecular dynamics simulations

The initial structures and all-atomistic (AA) force field (FF) parameters for the protonated and deprotonated forms of ALC-0315 were obtained from Petra Čechová et al. (2024) [1], while those for protonated and deprotonated MC3 were generated using CHARMM-GUI [2-5]. For each of the four ionizable lipids (ILs), a single molecule was placed in a cubic simulation box of $4.5 \times 4.5 \times 4.5$ nm^3, solvated with TIP3P water, and neutralized with the appropriate counterions. Energy minimization was performed using the steepest-descent algorithm [6] until the maximum force fell below 100 kJ mol^{-1} nm^{-1}. Subsequently, each system was equilibrated for 250 ps under NPT conditions, followed by a 500 ns production run. All AA simulations were carried out using GROMACS 2024 [7] under periodic boundary conditions. A 2 fs time step was employed, and all covalent bonds were constrained using the LINCS algorithm. Van der Waals interactions were computed with a cutoff of 1.4 nm with long-range dispersion corrections applied. Electrostatic interactions were treated using the reaction-field method with a dielectric constant of 80 and a real-space cutoff of 1.4 nm. The temperature was maintained at 298 K using the Nosé–Hoover thermostat with a relaxation time of 1 ps, and the pressure was controlled isotropically at 1 bar using the Parrinello–Rahman barostat with a relaxation time of 5 ps and a compressibility of 5×10^{-5} bar^{-1}.

CG mapping and simulation protocol

The CGbuilder tool [8] was used to convert the AA structures of the ILs into their CG representations. Based on the underlying AA chemical groups, clusters of three to five heavy atoms were mapped onto individual Martini-style beads (Figure S5). Each bead type was assigned according to the Martini 3 force-field definitions [9]. To determine the equilibrium bond lengths and angles for the CG topologies, the 500 ns AA trajectories were mapped onto

the corresponding CG representations, and snapshots were extracted every 20 ps. The average bond lengths and angles from these mapped trajectories were used as the equilibrium values in the CG model. The remaining bonded parameters—specifically the force constants for bonds and angles—were optimized iteratively. An initial set of force constants was assigned arbitrarily, and CG simulations were performed to generate CG trajectories. The resulting distributions of CG bond lengths and angles were then compared with those obtained from the mapped AA trajectories. The force constants were iteratively refined until the CG distributions closely reproduced the AA reference distributions (Figures S6 and S7).

For CG simulations, a single CG lipid molecule was placed in a cubic simulation box of $4.5 \times 4.5 \times 4.5$ nm^3, solvated with standard Martini water particles, and neutralized with counterions. Energy minimization was performed using the steepest-descent method with the same convergence criterion as in the AA simulations. Each system was equilibrated for 500 ps under NPT conditions using a 20 fs time step, followed by a 750 ns production run. CG molecular dynamics simulations using the Martini 3 force field together with the newly developed IL parameters were performed with GROMACS 2024 under periodic boundary conditions. Nonbonded interactions were treated using the Verlet cutoff scheme. Lennard–Jones and short-range electrostatic interactions were truncated at 1.1 nm and shifted to zero using the potential-shift-Verlet modifier. Temperature was maintained at 298 K using the velocity-rescale thermostat with a relaxation time of 1 ps, and pressure was controlled isotropically at 1 bar using the Parrinello–Rahman barostat with a relaxation time of 12 ps and a compressibility of 3×10^{-4} per bar.

To reproduce the procedures described above, all structures, input files, parameters, topologies, and scripts required for the AA and CG MD simulations are provided in our GitHub repository (https://github.com/mogam-ai/LNP_CG-MD (accessed on 18 November 2025)).

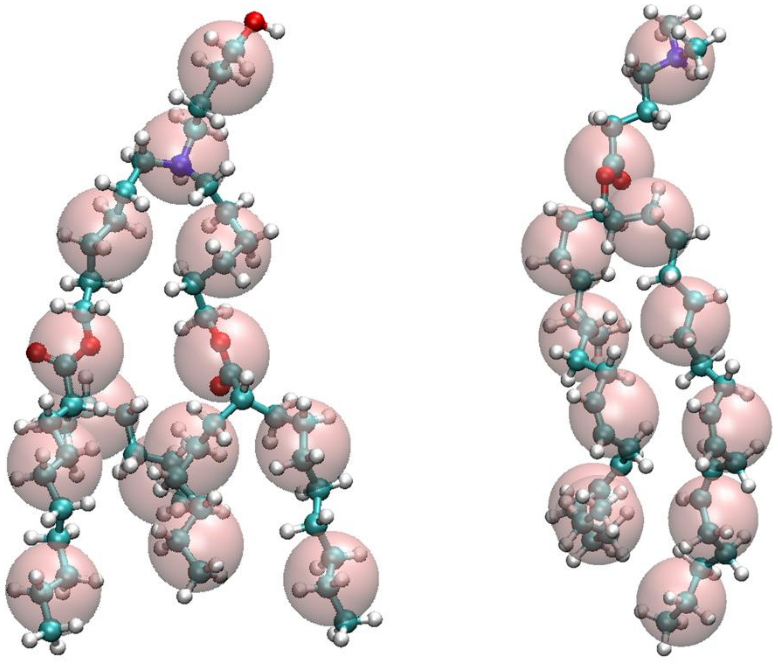

Figure S5. Mapping of corresponding CG beads based on the initial all-atom structures of ALC-0315 and MC3.

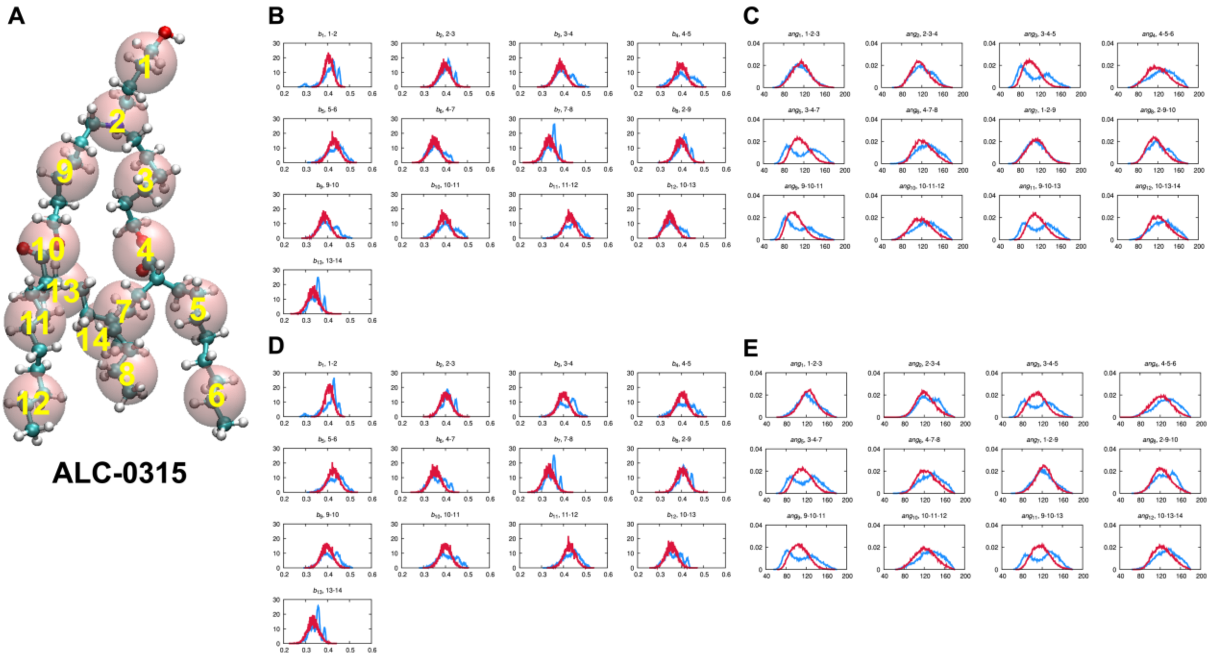

Figure S6. (A) Mapping of the CG beads onto the initial AA structure of ALC-0315 with numerical labels. (B, C) Comparison of bond length (B) and angle distributions (C) between AA MD simulations (blue) and CG MD simulations (red) for the deprotonated ALC-0315. (D, F) Comparison of bond length (D) and angle distributions (F) between AA MD simulations (blue) and CG MD simulations (red) for the protonated ALC-0315. AA MD simulations were conducted for 500 ns, yielding bond and angle distributions from 250,001 snapshots taken every 20 ps. CG MD simulations, using optimal CG FF parameters, were performed for 750 ns, generating corresponding distributions from 250,001 snapshots taken every 30 ps.

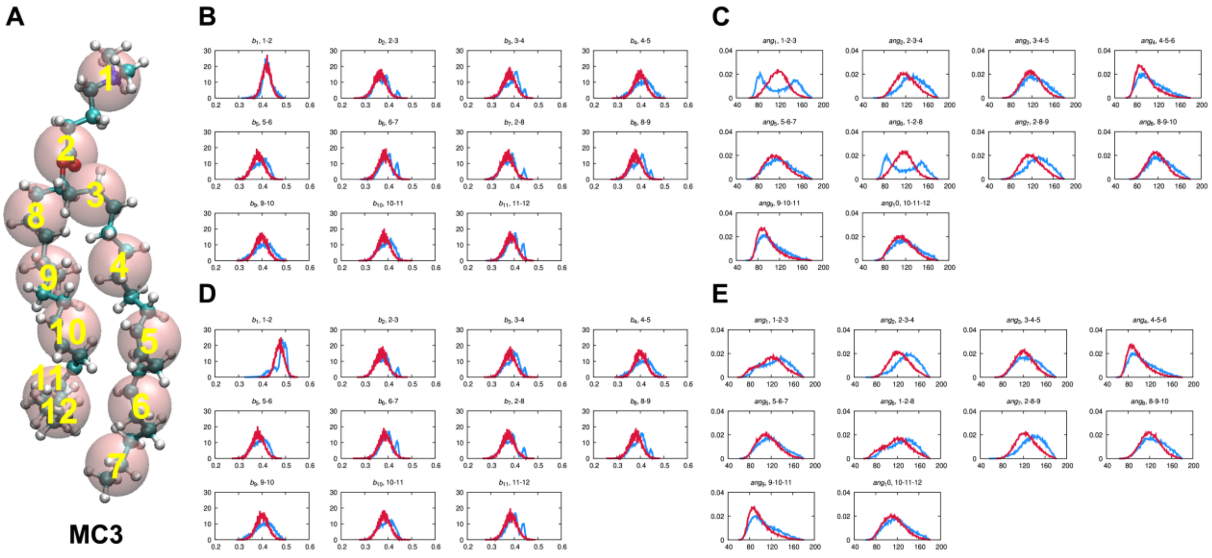

Figure S7. (A) Mapping of CG beads onto the initial AA structure of MC3 with numerical labels. (B, C) Comparison of bond lengths (B) and angle distributions (C) between AA MD simulations (blue) and CG MD simulations (red) for deprotonated MC3. (D, F) Comparison of bond lengths (D) and angle distributions (F) between AA MD simulations (blue) and CG MD simulations (red) for protonated MC3. AA MD simulations were conducted for 500 ns, producing bond and angle distributions from 250,001 snapshots extracted every 20 ps. CG MD simulations, with optimal CG FF parameters, were performed for 750 ns, generating corresponding distributions from 250,001 snapshots extracted every 30 ps.

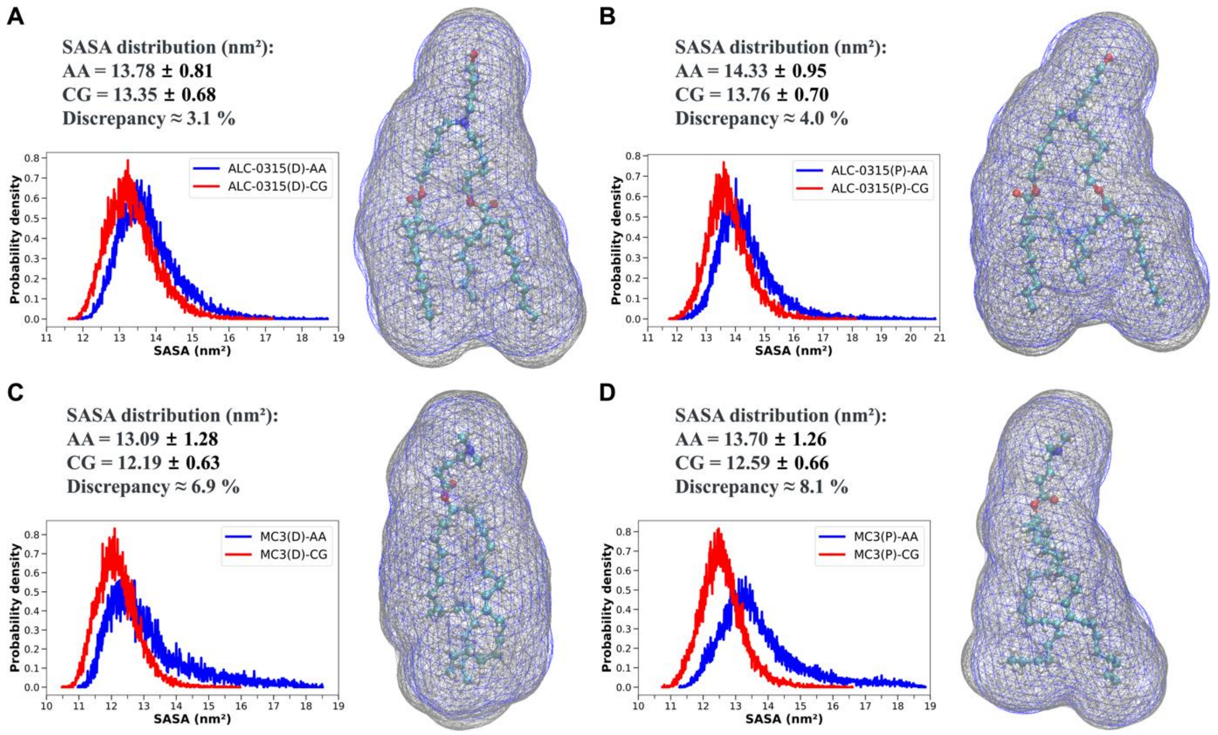

Figure S8. Comparison of solvent-accessible surface area (SASA) distributions and Connolly surfaces between CG and AA models. (A) SASA distributions of ALC-0315 (D) from AA (blue) and CG (red) models, indicating average values and discrepancies. Energy-minimized AA structures are shown overlaid with Connolly surfaces of AA (gray) and CG (blue) models. (B) ALC-0315 (P), (C) MC3 (D), and (D) MC3 (P). In all cases, the discrepancy in average SASA values between AA and CG models is below 10%, demonstrating acceptable agreement [10]. The close overlay of Connolly surfaces further indicates that the CG force field parameters effectively reproduce the molecular shape and solvent-accessible surface.

Table S7. Density comparison between AA and CG models.

Molecule	AA (kg/m³)	CG (kg/m³)	Discrepancy (%)
ALC-0315(D)	984.9 ± 13.5	987.0 ± 8.2	0.21
ALC-0315(P)	985.6 ± 13.5	988.3 ± 8.2	0.27
MC3(D)	1014.0 ± 0.06	986.9 ± 8.2	2.67
MC3(P)	1015.1 ± 13.5	987.8 ± 8.2	2.69

Table S8. Radius of gyration comparison between AA and CG models.

Molecule	AA (nm)	CG (nm)	Discrepancy (%)
ALC-0315(D)	0.56 ± 0.04	0.54 ± 0.03	3.57
ALC-0315(P)	0.58 ± 0.04	0.56 ± 0.03	3.45
MC3(D)	0.58 ± 0.08	0.53 ± 0.04	8.62
MC3(P)	0.60 ± 0.08	0.55 ± 0.04	8.33

Table S9. Radial distribution deviation between AA and CG models.

Molecule	RMS Δg*	Mean |Δg|	Max |Δg|
ALC-0315(D)	0.196	0.118	0.853
ALC-0315(P)	0.207	0.123	0.909
MC3(D)	0.205	0.122	0.851
MC3(P)	0.215	0.128	0.921

* RMS Δg values are dimensionless.

Supporting Reference

1. Cechova, P., et al., *Mechanistic insights into interactions between ionizable lipid nanodroplets and biomembranes.* J Biomol Struct Dyn, 2024: p. 1-11.
2. Jo, S., et al., *CHARMM-GUI: a web-based graphical user interface for CHARMM.* J Comput Chem, 2008. **29**(11): p. 1859-65.
3. Brooks, B.R., et al., *CHARMM: the biomolecular simulation program.* J Comput Chem, 2009. **30**(10): p. 1545-614.
4. Lee, J., et al., *CHARMM-GUI Input Generator for NAMD, GROMACS, AMBER, OpenMM, and CHARMM/OpenMM Simulations Using the CHARMM36 Additive Force Field.* J Chem Theory Comput, 2016. **12**(1): p. 405-13.
5. Park, S., et al., *CHARMM-GUI Membrane Builder for Lipid Nanoparticles with Ionizable Cationic Lipids and PEGylated Lipids.* J Chem Inf Model, 2021. **61**(10): p. 5192-5202.
6. Press, W.H.T., S.A.; Vetterling, W.T.; Flannery, B.P., *Numerical recipes 3rd edition: The art of scientific computing*. 2007, Cambridge university press.
7. Abraham M, A.A., Basov V, Bergh C, Briand E, Brown A, et al. *GROMACS 2024.4 Manual*. 2024; Available from: https://doi.org/10.5281/zenodo.14016613.
8. Barnoud, J.; Available from: https://github.com/jbarnoud/cgbuilder.
9. Souza, P.C.T., et al., *Martini 3: a general purpose force field for coarse-grained molecular dynamics.* Nat Methods, 2021. **18**(4): p. 382-388.
10. Alessandri, R., et al., *Martini 3 Coarse-Grained Force Field: Small Molecules.* Advanced Theory and Simulations, 2022. **5**(1): p. 2100391.
